# A New Scale Factor Adjustment Method for Magnetic Force Feedback Accelerometer

**DOI:** 10.3390/s17112471

**Published:** 2017-10-27

**Authors:** Xiangqing Huang, Zhongguang Deng, Yafei Xie, Zhu Li, Ji Fan, Liangcheng Tu

**Affiliations:** 1 MOE Key Laboratory of Fundamental Physical Quantities Measurement & Hubei Key Laboratory of Gravitation and Quantum Physics, School of Physics, Huazhong University of Science and Technology, Wuhan 430074, China; hxq160@hust.edu.cn (X.H.); dzg_109@hust.edu.cn (Z.D.); xieyaphe@hust.edu.cn (Y.X.); fanji@hust.edu.cn (J.F.); 2 School of Physics and Astronomy, Sun Yat-sen University, Guangzhou 510275, China; lizhu@sysu.edu.cn; 3 Institute of Geophysics, Huazhong University of Science and Technology, Wuhan 430074, China

**Keywords:** accelerometer, adjustment of scale factor, magnetic force feedback, gravity gradient instrument

## Abstract

A new and simple method to adjust the scale factor of a magnetic force feedback accelerometer is presented, which could be used in developing a rotating accelerometer gravity gradient instrument (GGI). Adjusting and matching the acceleration-to-current transfer function of the four accelerometers automatically is one of the basic and necessary technologies for rejecting the common mode accelerations in the development of GGI. In order to adjust the scale factor of the magnetic force rebalance accelerometer, an external current is injected and combined with the normal feedback current; they are then applied together to the torque coil of the magnetic actuator. The injected current could be varied proportionally according to the external adjustment needs, and the change in the acceleration-to-current transfer function then realized dynamically. The new adjustment method has the advantages of no extra assembly and ease of operation. Changes in the scale factors range from 33% smaller to 100% larger are verified experimentally by adjusting the different external coefficients. The static noise of the used accelerometer is compared under conditions with and without the injecting current, and the experimental results find no change at the current noise level, which further confirms the validity of the presented method.

## 1. Introduction

Over the past few decades, moving-base gravity gradiometers have been recognized as a viable solution for geodetic surveys [1,2], resource exploration [2,3,4,5], and inertial navigation [6,7,8]. The basic configuration of the rotating gravity gradiometer instrument (GGI) is an accelerometer complement consisting of two pairs of accelerometers. Their sensitive axes are tangential to the rotary disk. Each pair of accelerometers is set diametrically opposite to the other, in order to reject the common-mode linear acceleration perpendicular to the rotating axis, and to double the gradient signal. The two pairs of accelerometers are mounted orthogonally to each other. Their summed outputs are subtracted to suppress the angular acceleration around the rotating axis, and to double the gradient signal again. However, the performance of common-mode acceleration suppression depends on the precise matching of the accelerometers [4,5,9,10]. In terms of the need to maintain consistency of the sensitivities of state-of-the-art accelerometers, lack of online adjustment makes it difficult to meet such strict requirements [4,10]. In addition, the matching of the accelerometers is difficult to maintain for an extended period because of material aging, temperature variance, and electronic component instabilities, etc. The dynamic adjustment method for each configured accelerometer is a basic and necessary technology for GGI development.

In our previous experiment, we developed a quartz-flexure accelerometer with a high-performance electrostatic servo-controlled actuator. The electrostatic accelerometer adjusts the scale factor by trimming the electrostatic bias voltages [10]. This method has the advantage of ease of operation, and no extra trimming assembly process is needed. However, the input range of the electrostatic accelerometer is about 10 mg (1 g ≈ 9.8 m/s^2^), which is too small for a full tensor gradient measurement. When considering the full tensor measurement application, using magnetic force to rebalance the accelerometer with a large measuring range might be a better choice. The modified Model VII accelerometer used in Bell’s GGI is one specific application [4,5]. To adjust the scale factor dynamically, the accelerometer is disassembled, and an additional trimming coil is wound around the permanent magnet [11,12]. An appropriate current is calculated, which flows through the trimming coil to change the strength of the magnetic field acting on the torque coil. Thus, the scale factor can be adjusted. However, imperfect assembly will cause more nonlinear and cross-coupling effects, and the current in the trimming coil may cause extra unwanted heat effects, which will affect the relative permeability of the magnet. Hence, the stability of the magnetic field in the feedback loop will deteriorate.

By combining the advantages of the aforementioned methods, we propose a scale factor trimming method for a magnetic force rebalance accelerometer. An additional trimming current proportional to the feedback current is injected into the torque coil. The scale factor varies with that proportional coefficient, and the calibration results verify the validity of the trimming method. The method trims the feedback current instead of changing the strength of the magnet; hence, it needs no extra assembly and is easy to operate.

## 2. The Principle of the Scale Factor Adjustment Method by Trimming the Feedback Current

**Figure 1:**
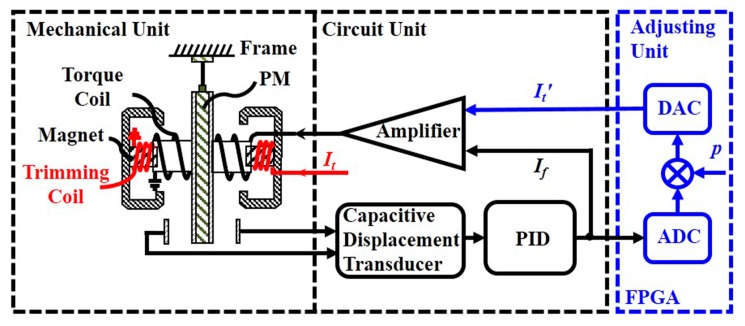
The diagram of a magnetic force rebalance accelerometer with the scale factor trimming. The red signal flow is the Bell’s scale factor adjusting method with trimming coil, while the blue signal flow is the new method with the trimming feedback current presented in this paper.

The diagram of a magnetic force rebalance accelerometer with scale factor trimming is illustrated in Figure 1. The magnetic force rebalance accelerometer consists of two units: the mechanical unit and the circuit unit. In the mechanical unit, the proof mass (PM) is suspended by a thin cantilever spring within a homogeneous magnetic field. The PM, which is coated with gold, works as a moving capacitive plate. The moving capacitive plate and the two fixed plates form a pair of differential capacitors. A couple of torque coils are distributed symmetrically on the PM. A permanent magnet is mounted in each fixed plate. Due to external acceleration, the PM will move with respect to the frame. The motion of the PM is sensed by the capacitive displacement transducer [10,13]. In the feedback path, a digital proportional integral differential (PID) controller provides a current *I_f_* related to the movement of the PM. The current *I_f_* flows through the torque coil and generates an equal and opposite force to compensate the movement. The related signal *I_f_* is therefore taken as the output of the accelerometer.

The force rebalance equation of the accelerometer is given by

(1) $$ma=NB_{0}LI_{f},$$

where *m* is the mass of the PM, *a* is the input acceleration, *N* is the number of turns of the torque coil, *B*_0_ is the strength of the permanent magnet, and *L* is the circumference of the torque coil. The physical parameters of the accelerometer appears in Table 1.

Thus, the transfer function from acceleration to feedback current, called the scale factor, can be derived to be

(2) $$K_{i0}=\frac{I_{f}}{a}=\frac{m}{NB_{0}L}.$$

In Bell’s scheme (red, Figure 1), an additional trimming coil is wound around the permanent magnet to adjust the scale factor. The current *I_t_* flowing through the trimming coil generates an additional magnetic strength ∆*B*. Hence, the magnetic field in which the torque coil acts is strengthened or weakened. Therefore, the force rebalance equation varies with the strength of the magnetic field, and the feedback force is given by

(3) $$F=N(B_{0}+\Delta B)LI_{f}.$$

Substituting the initial scale factor *K_i_*_0_ into the Equation (3), the trimmed scale factor *K_i_* has the following relationship

(4) $$K_{i}=\frac{I_{f}}{a}=\frac{K_{i0}}{1+\Delta B/B_{0}}.$$

From Equation (4), the scale factor varies with Δ*B*, thus it is adjusted by varying *I_t_*.

Bell’s scale factor adjustment method requires the disassembly of the mechanical unit so an additional trimming coil can be wound around the permanent magnet. To meet the gradient measurement requirement, the assembly of the accelerometer also needs to achieve a high degree of precision. The distance between the moving plate and fixed plates needs to be controlled with a precision greater than 100 μm [4]. Therefore, every single piece of the assembly process is very delicate. Imperfect assembly will generate nonlinear and cross-coupling output components, thus degrading the performance of the accelerometer.

By combining the advantages of the scale factor adjustment method of the electrostatic accelerometer used in our previous experiment [10] and the magnetic force rebalance accelerometer in Bell’s scheme [4,12], we can provide the adjustment principle as highlighted in the blue box in Figure 1. The output current *I_f_* is acquired with an Analog-Digital-Converter (ADC), multiplied by a scale proportionality coefficient *p* provided by the Field Programmable Gate Array (FPGA) according to the actual requirement, and subsequently transferred to analog current $I_{t}^{\prime }$ with a Digital–Analog Converter (DAC). The adjustment current $I_{t}^{\prime }$ and the feedback current *I_f_* are summed and injected into the torque coil to rebalance the PM. The adjusting current $I_{t}^{\prime }$ and the feedback current *I_f_* satisfy the relationship

(5) $$I_{t}^{\prime }=p\cdot I_{f}.$$

Thus, the total current *I_tot_* injected into the torque coil consists of two parts, *I_f_* and $I_{t}^{\prime }$, and they satisfy the relationship given by

(6) $$I_{tot}=I_{f}+I_{t}^{\prime }=I_{f}(1+p).$$

Then, the feedback force will be given by

(7) $$F=NB_{0}LI_{f}(1+p).$$

In this case, the scale factor $K_{i}^{\prime }$ and proportionality coefficient *p* satisfies the following relationship,

(8) $$K_{i}^{\prime }=\frac{I_{f}}{a}=\frac{K_{i0}}{1+p},$$

where *K_i_’* is the scale factor after trimming the feedback current. It can be seen from the expressions that the acceleration-to-current transfer function varies with the coefficient *p*. Comparing Equation (8) with Equation (4), we find that these two methods have similar formulas for the scale factor and are adjustable in common.

**Figure 2:**
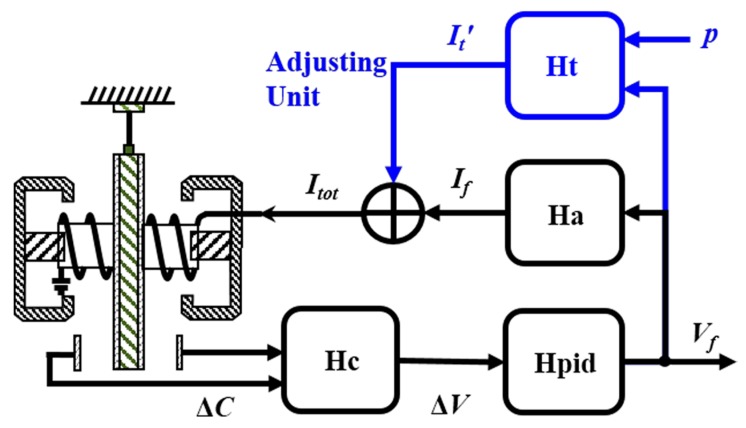
The block of the transfer function for the scale factor trimming. The value of the coefficient *p* can be provided numerically by FPGA according to the actual needs.

The block of the trimmed accelerometer, as shown in Figure 2, clearly demonstrates the changes in the circuit unit. The transfer functions of the capacitive displacement transducer, PID, the gain of the feedback voltage-to-current (V-I) amplifier and the adjusting V-I amplifier are written as *H*_c_, *H*_pid_, *H*_a_ and *H*_t_, respectively. The gain of *H*_t_ is adjustable by FPGA, and must satisfy the relationship *H*_t_ = *p* × *H*_a_ to ensure that Equation (5) is satisfied. After that, the adjusting current $I_{t}^{\prime }$ and the output current *I_f_* are summed as *I_tot_* and injected into the torque coil. To trim the scale factor of the accelerometer, all that needs to be done is to change the proportional coefficient *p* in FPGA. 

## 3. Experimental Verification

**Figure 3:**
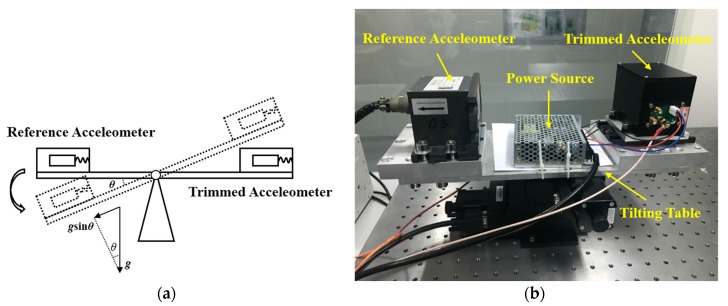
The calibration system. (**a**) Principle of the calibration with the tilting method. (**b**) The setup of the calibration system.

To verify the effectiveness of the scale factor adjustment method by trimming the feedback current, the accelerometers are calibrated by tilting their sensitive axes in a local gravity field with a commercial accelerometer as reference [13,14]. Both of the two accelerometers were mounted on a tilt table with their sensitive axes perpendicular to the tilt axis, as shown in Figure 3. The input axes of the accelerometers were changed relative to the gravity vector, with a series of different tilt angles *è_i_*. The adjustment unit (blue, Figure 1) consists of a digital circuit with a high-precision, 20-bit ADC AD7703, FPGA, and 20-bit DAC AD5791.

**Figure 4:**
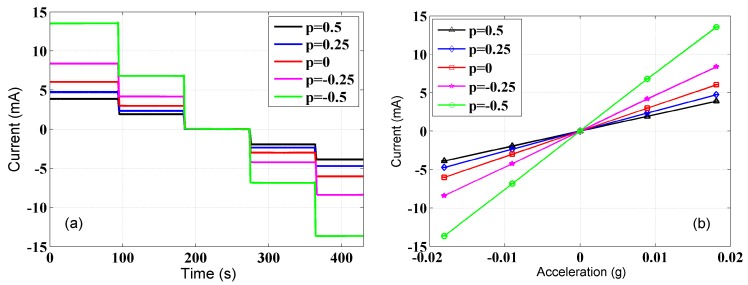
Calibration results of the scale factor of the trimmed accelerometer. (**a**) The output of the trimmed accelerometer with different proportional coefficient *p*. (**b**) The output of the trimmed accelerometer varies with changes in acceleration, and the slope of each line is the scale factor of the trimmed accelerometer corresponding to the proportional coefficient *p*. The standard deviation of each current step is at the order of 0.01 mA.

The data of the trimmed accelerometer and reference accelerometer were not collected until the system established temperature equilibrium with the environment and the sensors settled down in their positions. To calibrate the accelerometer, the input acceleration and the output current of the trimmed accelerometer need to be recorded at the same time. The input acceleration can be calculated based on the output voltage and the scale factor of the reference accelerometer given by *a_in_* = *V_out_*/*K*, where *V_out_* and *K* are the output voltage and the scale factor of the reference accelerometer respectively. The scale factor of the reference accelerometer used was 100 V/g. The tilt table was inclined an additional angle of about 9 mrad each time, and was kept still for about 90 s at each position to record the data. The calibration results are shown in Figure 4. The output currents of the trimmed accelerometer with different proportional coefficient *p* vary with time, as shown in Figure 4a. When taking the input acceleration derived from the output of the reference accelerometer as the *x*-axis and the output current of the trimmed accelerometer as the *y*-axis, accordingly, the slope of the line is the scale factor of the trimmed accelerometer, as shown in Figure 4b. 

**Figure 5:**
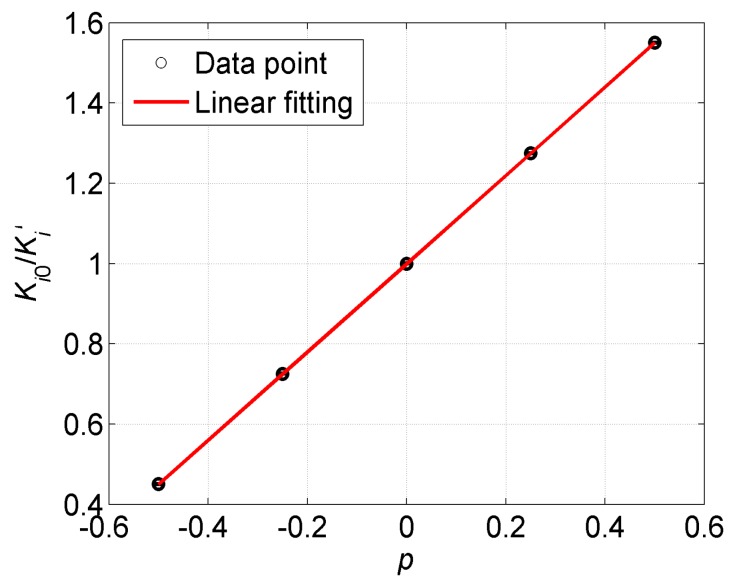
Linear fitting the ratio of $K_{i0}/K_{i}^{\prime }$ varying with the coefficient *p* according to Equation (8). The standard deviation of each point is at the order of 10^−4^.

To verify the new adjustment method directly, Equation (8) is transformed to $K_{i0}/K_{i}^{\prime }=1+p$, and we plot the ratio of $K_{i0}/K_{i}^{\prime }$ varying with the coefficient *p*, as shown in Figure 5. We find that the point where *p* = 0 corresponds to the initial scale factor *K_i_*_0_ of the accelerometer. By a least-squares linear fitting, the result is $K_{i0}/K_{i}^{\prime }=1.0001(2)+1.0001(6)p$, which results in good agreement with the expected value. The fitting results verify the proposed scale factor adjustment method. The changes of the scale factor are 33% smaller at *p* = 0.5 and 100% larger at *p* = −0.5 than the original one, which is in perfectly experimental agreement with Equation (8). In general cases of GGI, the scale factors of the accelerometers are able to achieve a consistency of ±10%, which corresponds to the range of *p* from +0.11 to −0.09. The scale factor balance loop would be able to calculate an appropriate coefficient *p* for each accelerometer in the GGI automatically [10]. Thus, this method would meet the requirement to expediently adjust the scale factor of the accelerometers in the development of the GGI.

**Figure 6:**
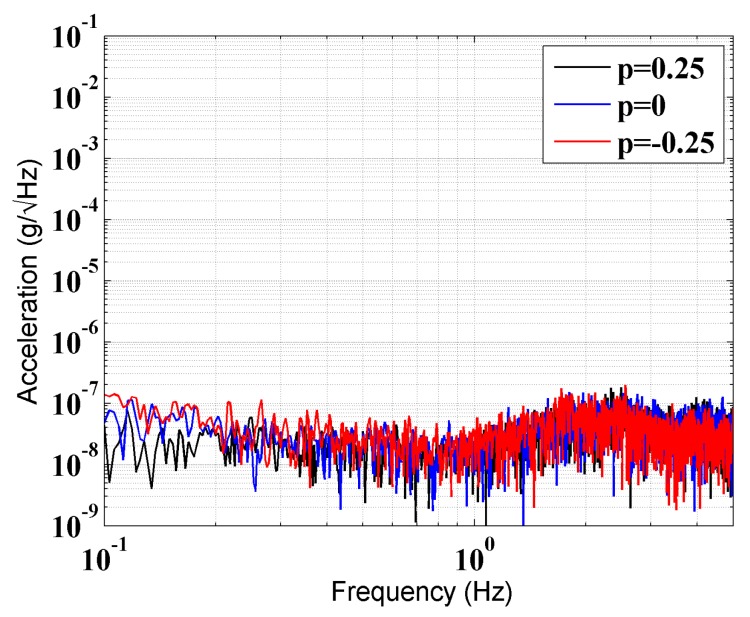
ASD of the trimmed accelerometer with three different coefficients *p*.

The noise level of the accelerometer is one of the key features of the GGI. However, the adjustment unit may introduce additional noise into the close-loop transfer function of the accelerometer. To determine whether the adjustment unit would deteriorate the noise level of the accelerometer, a static noise test was done. The trimmed accelerometer was mounted in our cave laboratory, where the vibration of the floor is relatively low, and the fluctuation of the temperature is lower than 0.03 K/day [15]. The acceleration spectral density (ASD) with different *p* was as shown in Figure 6. No obvious change in the noise level was observed in the frequency range from 0.2 to 1 Hz, in which the accelerometers would be kept rotating in the GGI. 

## 4. Conclusions

This paper proposes a new scale factor adjustment method by trimming the feedback current. The principle of the new adjustment method and the traditional method are equivalent. The calibration results verify the effectiveness of the new adjustment method. The static noise of the trimmed accelerometer does not change at the present noise level. Compared with the traditional adjustment method, the new adjustment method needs no extra assembly, is easy to operate, and could be used in the development of GGI.

## Figures and Tables

**Table 1:** Physical parameters of the accelerometer.

Parameter	Design
Proof mass m (mg)	10
Effective area of the differential capacitive (mm^2^)	350
Gap between the plates (ìm)	86
Strength of the magnet (T)	0.25
